# A cluster-randomised controlled trial to assess the effectiveness and cost-effectiveness of a childhood obesity prevention programme delivered through schools, targeting 6–7 year old children: the WAVES study protocol

**DOI:** 10.1186/s12889-015-1800-8

**Published:** 2015-05-13

**Authors:** Peymane Adab, Miranda J Pallan, Emma R Lancashire, Karla Hemming, Emma Frew, Tania Griffin, Timothy Barrett, Raj Bhopal, Janet E Cade, Amanda Daley, Jonathan Deeks, Joan Duda, Ulf Ekelund, Paramjit Gill, Eleanor McGee, Jayne Parry, Sandra Passmore, Kar Keung Cheng

**Affiliations:** Health & Population Sciences, University of Birmingham, B15 2TT Birmingham, UK; School of Clinical and Experimental Medicine, University of Birmingham, Birmingham, UK; Edinburgh Migration, Ethnicity and Health Research Group, Usher Institute of Population Health Sciences and Informatics, The University of Edinburgh, Edinburgh, Scotland; Food Science and Nutrition, University of Leeds, Leeds, UK; School of Sport, Exercise and Rehabilitation Sciences, University of Birmingham, Birmingham, UK; Cambridge MRC Epidemiology Unit, Cambridge, UK; Norwegian School of Sport Sciences, Oslo, Norway; Birmingham Community Healthcare NHS Trust, Birmingham, UK; Services for Education, Birmingham, UK

**Keywords:** Cluster randomised controlled trial, Complex intervention, Childhood obesity prevention, Physical activity, Healthy eating, Cost-effectiveness

## Abstract

**Background:**

There is some evidence that school-based interventions are effective in preventing childhood obesity. However, longer term outcomes, equity of effects and cost-effectiveness of interventions have not been assessed.

The aim of this trial is to assess the clinical and cost-effectiveness of a multi-component intervention programme targeting the school and family environment through primary schools, in preventing obesity in 6–7 year old children, compared to usual practice.

**Methods:**

This cluster randomised controlled trial is set in 54 primary schools within the West Midlands, UK, including a multi-ethnic, socioeconomically diverse population of children aged 6–7 years.

The 12-month intervention consists of healthy diet and physical activity promotion. These include: activities to increase time spent doing physical activity within the school day, participation in the ‘Villa Vitality’ programme (a programme that is delivered by an iconic sporting institution (Aston Villa Football Club), which provides interactive learning opportunities for physical activity and healthy eating), healthy cooking skills workshops in school time for parents and children, and provision of information to families signposting local leisure opportunities. The primary (clinical) outcome is the difference in body mass index (BMI) z-scores between arms at 3 and 18 months post-intervention completion. Cost per Quality Adjusted Life Year (QALY) will also be assessed. The sample size estimate (1000 children split across 50 schools at follow-up) is based on 90% power to detect differences in BMI z-score of 0.25 (estimated ICC ≤ 0.04), assuming a correlation between baseline and follow-up BMI z-score of 0.9. Treatment effects will be examined using mixed model ANCOVA. Primary analysis will adjust for baseline BMI z-score, and secondary analysis will adjust for pre-specified baseline school and child level covariates.

**Discussion:**

The West Midlands ActiVe lifestyle and healthy Eating in School children (WAVES) study is the first trial that will examine the cost-effectiveness and long term outcomes of a childhood obesity prevention programme in a multi-ethnic population, with a sufficient sample size to detect clinically important differences in adiposity. The intervention was developed using the Medical Research Council framework for complex interventions, and outcomes are measured objectively, together with a comprehensive process evaluation.

**Trial registration:**

Current Controlled Trials ISRCTN97000586 (registered May 2010).

## Background

Childhood overweight and obesity is an ever increasing public health concern [1] which has serious health consequences in both child [2] and adult life [3]. Children as young as 7 years old, who are obese, are at higher risk of premature mortality in adulthood, compared to their normal weight counterparts [4] and from the age of 11 years, there is tracking of behaviours [5], such that over 50% of obese children become obese adults [6]. In England childhood obesity rates have increased over the last 20 years. A doubling of prevalence of obesity can be observed between the ages of 4 and 11 years (the primary school years) [7]. This is the time period of adiposity rebound, which occurs following a nadir in Body Mass Index (BMI) around the age of 5–6 years [8]. Thus the primary school years are a key time period for targeting interventions for the prevention of childhood obesity. In terms of settings, schools are an environment in which the majority of children spend a sustained period of time. They provide an infrastructure through which children and their parents can be identified and receive, both within and outside the curriculum, opportunities to learn about, practice and reinforce healthy lifestyle behaviours.

Several systematic reviews [9-11] have summarised the outcomes of previous childhood obesity prevention studies, undertaken in a variety of settings including school, community and family. The most up-to-date Cochrane review of trials, published in 2011, showed that school based interventions, particularly those targeting 6–12 year olds, are effective in reducing adiposity (mean effect size −0.15 for BMI z-score). However, there was much heterogeneity in intervention components and design and generally small sample sizes. Furthermore previous trials were poor at reporting process and implementation measures, rarely considered equity of effects in relation to sex, ethnicity or other subgroups, tended not to report longer term outcomes and seldom reported on adverse effects or costs.

### Development of a childhood obesity prevention programme

The Birmingham healthy Eating and Active lifestyle for CHildren Study (BEACHeS) was funded by the UK National Prevention Research Initiative and took place from 2006 to 2009. The study used the early phases of the UK Medical Council Research framework for complex intervention development and evaluation [12] to develop a childhood obesity prevention programme aimed at children aged 6–8 years, and tested its feasibility and acceptability in an exploratory trial. A number of different methodologies were employed and iteratively combined in the theoretical and modelling phases of intervention development [13]. These included a review of childhood obesity prevention evidence, focus groups with key stakeholders to explore their views of the causes of childhood obesity [14] and their perceptions of preventive approaches, consultation with a group of professionals, and a review of existing local resources and national policy. The Analysis Grid for Environments Linked to Obesity (ANGELO framework) [15] was applied during the development process to ensure the intervention addressed all relevant environmental dimensions. The initial programme consisted of two broad strands: increasing children’s physical activity levels through school, and family healthy behaviour skills (food preparation and physical activity) through activity based learning. The programme was tested and further refined through a feasibility study involving eight primary schools in Birmingham, UK [16], which provided justification for a more definitive evaluation of the intervention.

In this paper we describe the study protocol for the definitive evaluation (a cluster randomised controlled trial); the West Midlands ActiVe lifestyle and healthy Eating in School children (WAVES) study, funded by the UK National Institute for Health Research (NIHR) Health Technology Assessment programme.

#### Trial aims and objectives

The main aim is to assess the clinical and cost-effectiveness of the 12-month childhood obesity prevention intervention programme, developed and refined in the BEACHeS study, using usual practice in primary schools as the comparator. Intervention effects will be examined at 3 and 18 months post-intervention completion. Cost-effectiveness of the intervention will be assessed from a societal perspective. In addition, differences in intermediate and final outcomes will be explored by sex, ethnicity, socioeconomic status and weight status. We will also use a variety of methods to describe the implementation of, and adherence to, intervention components [17].

#### Trial design and overview

**Figure 1:**
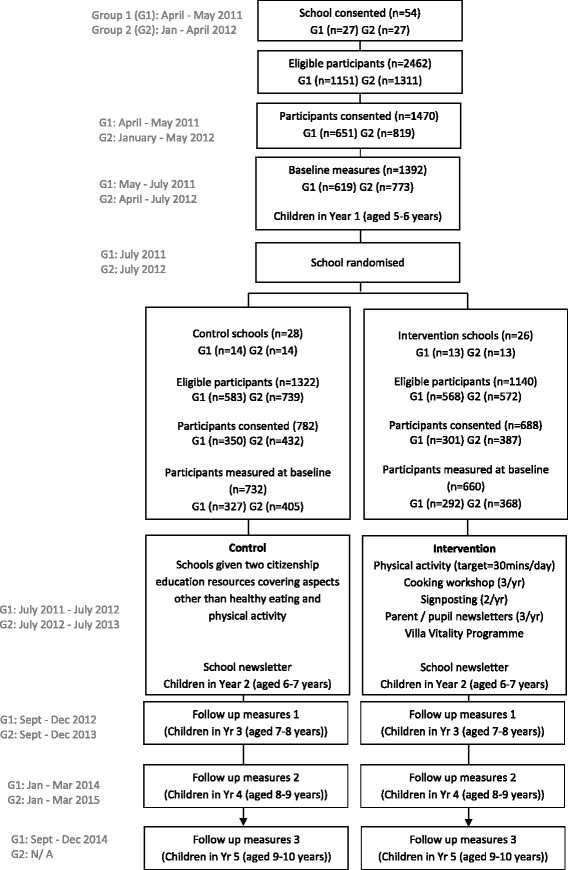
Study design and the flow of study participants through the WAVES study.

The WAVES study is a cluster-randomised controlled trial. Primary schools (n = 54) are recruited from a multi-ethnic population within the West Midlands, UK. Randomization is at the level of the cluster (school). Data are collected at both the cluster (school) and within cluster (individual pupils and their parents) level. To test the effect of the intervention, a range of anthropometric and psychological data are collected (described in detail later) on children within participating schools. Baseline measures are undertaken when the participating children are in Year 1 (April to July; aged 5 to 6 years). Schools are then randomly allocated to either the usual practice or intervention arm. Schools in the intervention arm are asked to implement a 12 month, multifaceted intervention programme (details below) when children are in Year 2 (aged 6 to 7 years). The programme includes physical activity and dietary components, targeting the school and family environments and aims to help children maintain a healthy weight, thereby preventing overweight/obesity. Due to practical considerations, half the schools (Group 1) are recruited to commence the study in the 2011/12 school year and the remainder (Group 2) in the 2012/13 school year. First follow-up measures are undertaken immediately after the intervention year (September to December, when the children are in Year 3; aged 7 to 8 years) and second follow-up measures are undertaken 18 months post intervention (January to March, when the children are in Year 4; aged 8 to 9 years). Group 1 schools receive a third set of follow-up measures 27 months post intervention completion (September to December when in Year 5; aged 9 to 10 years). A summary of the study design and timelines is shown in Figure 1.

NHS Research Ethics approval for the trial was obtained from the Black Country Research Ethics Committee (NHS REC no.10/H1202/69). The trial was registered in May 2010 (ISRCTN97000586).

## Methods

### Study setting and participant eligibility

All state primary schools in the West Midlands (UK) which included school years 1 to 5 (children aged 5 to 10 years) and that were within a 35 mile radius of the University of Birmingham were eligible for inclusion (n = 980). To ensure sufficient representation in the sample to enable sub-group analysis by minority ethnic group, school populations were stratified by ethnic mix including White, South Asian (comprising Indian, Bangladeshi and Pakistani) and Black (including African and Caribbean), with the remainder being classified as “Other” ethnicity. School populations were dichotomised as being in the top 80th percentile in terms of South Asian or Black pupil representation, or not. The sampling strategy used a weighted random sample so that schools with a higher minority ethnic population (in top 80th percentile for South Asian or Black) had an increased chance of being sampled with a ratio of 3:1. Given the relatively large number of clusters (>50) the sampling strategy was also balanced to take account of three other important factors to ensure a range of characteristics are represented. These were: proportion of children eligible for free school meals (FSM) as an indicator of socio-economic make-up of the pupils, school size and urban/rural location of the school. Using this method, 200 schools were selected and ordered using a random number generator. Of these, 7 were excluded as they did not fit the eligibility criteria. The remaining schools were sequentially invited to participate, and 148 were approached until the required sample size (54 schools) was achieved. Of the 148 schools approached, 4 did not respond and 90 declined to participate.

### Exclusion criteria

Schools with fewer than 17 pupils in the relevant year group (minimum cluster size), or those that were in special measures (status applied by the Office for Standards in Education when it considers that a school fails to supply an acceptable level of education and appears to lack the leadership capacity necessary to secure improvements) were excluded.

### School recruitment process

Schools were approached by letter, followed by a phone call and a visit to interested schools. All participating schools (control and intervention) receive a financial reimbursement (£190) following each period of pupil measurement to compensate for staff time spent on the study. Regular newsletters are sent to participating schools to maintain engagement.

### Recruitment of study participants

All Year 1 pupils (aged 5 to 6 years) in participating schools were eligible to take part. An invitation letter, information leaflet and consent form were distributed through schools to parents/carers of eligible pupils.

### Trial intervention

The WAVES study intervention programme has four components (outlined below) delivered over 12 months. There is also a termly family newsletter to reinforce the messages delivered through the various components. The schools are used as the platform for disseminating information, targeting intervention children and their families, and as the venue for some of the intervention components. Each component has fixed parameters as well as elements that allow tailoring to specific populations, enabling schools to adapt the delivery by taking account of local circumstances. Relevant school staff members are provided with a manual and a short training session on delivering the intervention. Follow-up support for intervention delivery is provided by research staff in the first few weeks. No further support is provided for the remaining intervention period.

Schools in the intervention arm receive reimbursement (£380) to cover costs incurred through their involvement with the intervention (such as staff cover for teacher training).

#### Component 1: Structured physical activity opportunities during the school day

The overall aim of this component is to increase physical activity opportunities within the school day, with a target for children to achieve an additional 30 minutes of moderate to vigorous physical activity (MVPA) per day. Teachers can select two from a choice of four activity programmes which are available in the UK market; ‘Activate’ [18], ‘Positive Play’ [19], ‘Take 10’ [20] or ‘Wake Up Shake Up’ [21]. These programmes were selected as they could be tailored to each school setting and incorporate a range of classroom and playground based routines to help children be active in a school environment with minimal disruption to the regular school day.

#### Component 2: Cooking skills workshops for children and parents

The aim of this component is to increase healthy eating knowledge and improve food preparation skills of parents and children. A series of three workshops, designed by research nutritionists for children and their parents or carers, is delivered by school staff. Each workshop was piloted (with 6 to 8 children aged 6–7 years and their parents) and the content and format modified as necessary prior to completion. Relevant school staff members are invited to attend interactive training on the content and delivery of the workshops, where they are provided with all relevant materials (including lesson plans and presentation slides) and participate in a practical session. The workshops, which are intended to be delivered once per term through the school year, focus on ‘breakfast’, ‘lunch and snacks’ and ‘evening meal’. Key messages are consistently included across all sessions to increase fruit, vegetable and fibre intakes, and decrease fat and sugar intakes. Each workshop is preceded by three 10 minute lessons for the children in class time to prepare them for the topics to be covered. During the workshop an interactive educational session is followed by practical food preparation, where children work with their parents to prepare healthy food that they can eat together. Written information emphasising key messages is given to parents and carers to take home after the workshops.

#### Component 3: Signposting

The aim of this component is to increase participation in physical activity out of school hours. Children are given two information sheets to take home, signposting opportunities and facilities for them and their family to be physically active. Following baseline measures, children in intervention schools are given a brightly coloured information sheet which highlights the UK government recommendation of at least 60 minutes of moderate to vigorous physical activity a day [22], and uses motivational statements (such as encouraging goal setting) as well as information and ideas for how children and their families could be active over the summer holidays. The focus is on everyday opportunities such as walking and physical activities that could be done in the home. At the beginning of the following term, they are given more specific information, which again highlights the 60 minutes of activity message, and gives details of clubs, leisure centres, parks and other opportunities suitable for families with young children to undertake physical activity within close proximity of their school.

#### Component 4: Villa Vitality

The aim of this component is to promote healthy lifestyle messages through an iconic sports institution and its staff. Villa Vitality (VV) is a programme run at Aston Villa Football Club (AVFC; a premier league English football club), focusing on promoting healthy eating and physical activity through interactive sessions delivered at AVFC. The programme, originally designed for older children, has been adapted for the WAVES study by collaboration between VV staff and the research team. All the messages delivered as part of the programme are consistent with the other components of the WAVES study intervention. The revised programme was piloted with a sample of Year 2 children (n = 60) before implementation in the study intervention arm.

The VV programme involves two day trips to AVFC, six weeks apart. The children participate in a range of activities during these days. These include: physical activity games and ball skills, two nutrition education sessions, dance mats, preparing a meal in the VV kitchen, a tour of the stadium and a session in the VV radio studio. During the intervening 6 weeks, the children are encouraged to participate in weekly health challenges (achieve 60 minutes of activity every day, swap a snack, drink more water, eat a healthy breakfast every day, eat 5 portions of fruit and veg every day and cook a healthy family meal), and undertake a class project (to produce a song, story or poem about healthy living for recording during their session in the VV radio studio). The children also receive a 60 minute physical activity session run at school by an Aston Villa Football Academy coach. During this visit the coach also reviews progress in relation to both the class project and the weekly challenges.

### Comparator

Schools in the usual practice (control) arm are sent citizenship education resources [23] to use as they wish (the topics of healthy eating and physical activity are intentionally avoided). No other active intervention is offered. These schools continue with any ongoing health related activities.

### Method of random allocation and blinding

A blocked balancing algorithm is used to randomise schools to either the intervention or control arm [24,25]. This algorithm randomly selects one of a number of allocation designs which minimise the imbalance between covariate means. The covariates included are percentage of pupils within the school eligible for free school meals; percentage of South Asian pupils within the school; percentage of Black pupils within the school; percentage of White pupils within the school; and number of pupils within the school. Randomisation is undertaken after baseline measurements, and participating schools are then informed of allocation.

### Outcome measures

For clinical effectiveness, the primary outcome is the difference in body mass index (BMI) z-scores (using the UK 1990 BMI reference curves for children [26]) between arms at 3- and 18-month follow-up post-intervention completion. Secondary outcomes include: i) anthropometric measures: percentage overweight and obese (defined as a BMI greater than the 85th percentile on the UK 1990 reference charts for BMI centiles for boys and girls), skinfold thickness at 5 sites (biceps, triceps, thigh, suprailiac and subscapular), waist circumference and percentage body fat; ii) blood pressure; iii) dietary energy (kJ per kg body weight per day), fat, sugar, fibre (g/day), and fruit and vegetable intake (g/day and portions); iv) physical activity energy expenditure (kJ per kg body weight per day), and time spent doing sedentary, light, moderate and vigorous intensity activity (min/day), v) psychosocial outcomes to assess the wider effects of the intervention, including benefits and potential harms: health related quality of life and body dissatisfaction, and vi) longer term clinical effectiveness at 27 months post intervention in Group 1 schools.

For cost-effectiveness analysis, the primary outcome is cost per Quality-Adjusted Life Year (QALY). Other analyses will include cost per effectiveness outcomes such as change in BMI z-score and change in proportion of overweight/obese. A longer term model-based evaluation will predict cost-per-QALY outcomes over a lifetime by linking a change in weight status in childhood to future health outcomes in adulthood.

### Data collection methods

Pupils’ date of birth, sex, ethnicity and postcode (to derive a proxy measure for deprivation) data are obtained from parent questionnaire, or if not available, from school records. Assessments are undertaken in school by trained research staff, using standardised procedures (available on request) and validated instruments at baseline and follow-up time points. In addition, parents of participating children are asked to complete questionnaires at each time point. These cover questions on child and parent demographics, dietary, sedentary, physical and sleep activity habits, home food environment, perceived neighbourhood environment and proximity to food and leisure facilities, family cooking habits and participation in leisure activities.

### Anthropometric measures

All measurements are undertaken barefoot and in light clothing. Standing height is measured at least twice (with a third measure if difference is >0.4 cm) with a Leicester Height Measure. Weight and body fat percentage are measured with a Tanita bioimpedance monitor (Tanita SC-331S; Tanita Corporation., Tokyo, Japan). Waist, arm and thigh circumference are measured at least twice (with a third measure if difference is >0.4 cm, 0.2 cm or 0.2 cm, respectively) using a non-stretch tape-measure. Skinfold thickness at five sites (biceps, triceps, subscapular, suprailiac and thigh) are measured at least twice (with a third measure if difference is >0.4 cm) on the non-dominant side, using a Holtain Tanner/Whitehouse Skinfold Caliper (Holtain Ltd., UK).

Dietary assessment is undertaken using a validated [27] simple tick list, the Child and Diet Evaluation Tool (CADET), which is completed by researchers (in school) and parents (out of school) over a 24 hour period. The tool enables estimation of total energy, macro and micro-nutrient intake.

Physical activity energy expenditure and its sub-dimensions (i.e. time spent sedentary and in light, moderate and vigorous intensity activity) is assessed objectively over a 5 day period (including a weekend) using a monitor that combines heart rate and accelerometry (Actiheart, Cambridge Neurotechnology Ltd, Papworth, UK), which has excellent technical validity and reliability [28] and has been validated in young children [29].

### Blood pressure

Blood pressure is measured using clinically validated, automated, oscillometric BP monitors (BpTRU BPM-100, British Columbia, Canada) [30], with the appropriate cuff-size used for each child. After 3 minutes seated-rest, two readings are taken with a 3 minute rest-interval between each. A third measurement is taken if an error reading occurs, or if one of the values is outside the normal range.

### Other measures

Psychosocial measure are collected through researcher administered questionnaires to children. Quality of life is measured using the Pediatric Quality of Life Inventory (PedsQL) [31]; social acceptance is measured using the relevant domain from the Kidscreen-52 health questionnaire for children and young people [32] and body image is assessed using the Children’s Body Image Scale [33]. The pediatric preference-based utility instrument, Child Health Utility 9D [34] is also completed to inform the economic evaluation.

#### School level data

Data on participating schools are collected through a questionnaire administered to head teachers or a nominated representative. Information requested includes details on school food and physical activity policies and any relevant initiatives or programmes delivered through school.

### Process evaluation

Implementation fidelity is assessed throughout the intervention year using a range of methods including direct observation, logbooks, parent and school staff questionnaires, research staff experiences and qualitative evaluation. The methods are described in detail elsewhere [17].

### Justification of sample size

Sample size calculation is based on the primary outcome (BMI z-score). Further calculations were also performed to estimate power for the secondary outcome of percentage of children overweight or obese. Planned analysis of the WAVES study will compare outcomes for control and intervention schools at follow-up times, adjusting for baseline measurements. Therefore power calculations undertaken were based on repeated measures methods using estimates of correlation between before and after measurements. A modified version of the design effect [35] was used to estimate sample size and accommodate varying cluster sizes (using the estimated: mean cluster size (n = 25; SD = 23). For the primary outcome of BMI z-score, a follow-up sample size of 1000 children split across 50 schools gives the study greater than 90% power to detect a difference of 0.25 BMI z-score between intervention and comparator groups (equivalent to approximately 0.5 kg body weight for a 7-year old child) under all likely estimates of the intraclass correlation coefficient (ICC = 0 to 0.04, estimated correlation between before and after measures = 0.9 and estimated dropout rate = 20%). A change of 0.25 in BMI z-score has been shown to be associated with clinically detectable benefits in obese adolescents [36] and longitudinal studies demonstrate a linear relationship between BMI z-score in children as young as 7 and heart disease events in adulthood [4]. Under more conservative estimates for the ICC, this sample size would provide more than 80% power to detect a 0.125 difference in BMI z-score. A BMI z-score difference as low as 0.125 is the primary outcome of choice for other childhood obesity prevention trials [37]. Allowing for school drop-out (~8%), 54 schools were therefore invited to take part.

For the secondary outcome of percentage overweight/obese, this sample size (with an estimated correlation of before and after measures of 0.7 and an ICC of between 0 and 0.02) provides greater than 80% power to detect a difference in the change of proportion of children who are overweight/obese from baseline to follow-up in control compared to intervention schools of about 7% (exact value depends on baseline values). All power calculations were carried out in STATA using the clustersampsi function [38].

### Data quality and management

All study data are stored in a password-protected customised database, hosted by the University of Birmingham. Paper-based information is held in locked filing cabinets in the study office. For all data entry, a minimum 10% sample is checked to monitor error rates. Potential errors are identified and checked using a range of techniques. These include clinical and data-driven range checks, and cross validation between variables where a correlation would be expected and when the same information is obtained from different sources.

### Planned statistical analysis

Trial analyses will be undertaken after the second follow-up measures are completed and there will be no interim analyses.

The baseline pupil (including sex, ethnicity, deprivation [based on IMD scores derived from home postcode] anthropometric measures, dietary intake, physical activity levels, psychological variables) and school level characteristics (school size, ethnic mix of pupils and % eligible for FSM) will be summarised by control and intervention arms, using numbers and proportions, means and standard deviations or medians and inter-quartile ranges.

Analyses of outcomes will be by intention to treat. As randomisation will be at the school (cluster) level, appropriate statistical methods to account for the clustering within schools (detailed below) will be used in the analysis. Analysis of outcomes will be for both 3- and 18-month follow-up stages.

We will use a mixed model ANCOVA with follow-up outcome values as the dependent variable and baseline values and treatment arm as the independent variables, to investigate effectiveness. These will be fitted using mixed models in STATA to allow for clustering. We will allow for clustering at the school level and explore the possibility of allowing for an additional level of clustering at the class level.

The primary analysis will be adjusted for baseline values for all outcomes. Secondary analysis will additionally adjust for pre-specified baseline school and child level covariates. These will include school level factors which were used in the randomisation (school size, % pupils eligible for free school meals, ethnic mix of pupils) and pupil level factors (sex, baseline BMI z-score, ethnicity, deprivation from home postcode, baseline total energy intake and baseline total physical activity). We will not adjust for age as all children will be of a very similar age. We will adjust at the school and pupil level for both ethnicity and deprivation as the school population is expected to differ from the consented study population.

Outcomes are either binary (e.g. non-overweight vs. overweight), or continuous (e.g. BMI z-score or energy expenditure), and therefore either log or linear link functions will be used, with transformations where appropriate to accommodate any non-normality. All model assumptions will be checked. We will report both relative and absolute treatment effects.

The primary analysis will be a complete case analysis. However, missing data will be reported and associations between outcomes explored. Depending on the nature of these associations and the extent of the missing data, sensitivity analysis will be undertaken using multiple-imputation techniques.

The primary outcome and primary sub-group comparisons at both time points will be considered significant at the 5% level (and so 95% CIs reported); whereas other secondary outcomes will be deemed significant at the 1% level (and so 99% CIs reported). This difference in levels of significance, gives more weight to the primary outcomes.

#### Planned subgroup analyses

An examination of whether any difference in outcomes between control and intervention arms varies by sex, weight status at baseline, ethnic mix of the school and socio-economic factors will be undertaken. Within the intervention arm, we will also look at differences in outcome by fidelity of implementation (broadly classified as low, medium or high).

The significance of subgroup effects will be assessed by tests of interactions of covariates and the treatment effect. The study will have low power to detect all but the largest differences in subgroups.

### Economic evaluation

The economic evaluation will estimate the incremental cost and incremental benefit of the WAVES study intervention compared to usual current practice, from a NHS/educational service perspective. Additional wider perspectives, such as inclusion of family members, will be explored as part of a sensitivity analysis. A within-trial analysis will estimate the cost-effectiveness at 18 months assuming that the intervention is in a ‘steady state’ and thus will not include set up or implementation costs. A longer term analysis will estimate the cost effectiveness using a decision-analytic model.

The within-trial analysis will adopt a micro-costing approach to estimate the costs of each intervention component. Trial report forms and school logbooks will collect resource use information for staff time, materials, transport, and equipment, combined with unit cost data to estimate the incremental intervention mean cost per class, and per child. Sensitivity analysis will explore intervention fidelity, and the inclusion/exclusion of categories of cost e.g. family members, set up, implementation. Quality of life will be measured using the Child-Health Utility 9D instrument and expressed as QALYs. Cost-effectiveness will be measured using both the effectiveness outcomes (BMI z-score, proportion overweight/obese) and QALY outcomes.

The long-term cost effectiveness analysis will use a decision-analytic model to predict the cost-savings and outcomes from preventing overweight/obesity in childhood. Model parameters will be informed by a literature review and will map outcomes from childhood to adulthood. Extensive sensitivity analysis will be carried out, to test for the robustness of the conclusions to assumptions made in the modelling, and to sampling variation in the data used in the construction of the model. Costs and benefits will be discounted at the standard rate (3.5%).

### Trial status

The trial started recruitment of schools in January 2011, and of pupils from March 2011. Intervention delivery was completed in July 2013. Final follow-up measurements will be completed in April 2015. Data analysis will commence following data cleaning (after June 2015). The expected report date is November 2015.

## Discussion

To our knowledge, the WAVES study is the first trial of a childhood obesity prevention intervention that: has been developed using the MRC framework for complex interventions, tests both the clinical and cost-effectiveness of a school-based intervention and will have sufficient length of follow-up to examine longer term effects. The trial setting includes a diverse socioeconomic and multi-ethnic population to allow exploration of sub-group effects. There is also consideration of a wide range of outcomes, including psychosocial effects to monitor potential harm.

The trial will address some of the limitations identified in previous research [9], particularly including a sample size large enough to detect clinically significant differences in adiposity, use of an objective measure of physical activity, inclusion of cost effectiveness evaluation, a comprehensive process evaluation and assessment of longer term outcomes (all schools at 18 months and half the schools at 27 months post intervention completion).

Given the pragmatic and complex nature of the trial, it will not be possible to assess intervention efficacy directly or to disentangle the relative contribution of different intervention components to any observed outcomes. On the other hand, assessment of effectiveness in real settings facilitates future intervention roll-out and dissemination, should the intervention prove to be clinically cost-effective. Thus, the study has the potential to influence health and education policy in the UK and further afield.

The comprehensive process evaluation and detailed assessment of implementation alongside the trial will allow us to contextualise and explain the findings of the trial and inform future implementation. It will also allow us to perform analyses to explore the relationship between intervention implementation and outcomes, which has not been undertaken in previous childhood obesity prevention trials.

In addition to the findings of the trial, the study will also provide a large dataset on the weight status and other health indicators of a sub sample of multi-ethnic children in the West Midlands, which can be used to address other relevant research questions.

## Abbreviations

ANGELO: Analysis Grid for Environments Linked to Obesity
BEACHeS: Birmingham healthy Eating and Active lifestyle for CHildren Study
BMI: Body Mass Index
CADET: Child and Diet Evaluation Tool
FSM: Free school meals
ICC: Intraclass correlation coefficient
MRC: Medical Research Council
MVPA: Moderate to vigorous physical activity
NIHR: National Institute for Health Research
PedsQL: Pediatric Quality of Life Inventory
WAVES: West Midlands ActiVe lifestyles and healthy Eating in School children study
VV: Villa Vitality
